# Engaging youth in health and research in rural Cambodia: a qualitative study

**DOI:** 10.1080/16549716.2026.2684845

**Published:** 2026-06-15

**Authors:** Mom Ean, Lek Dysoley, Hem Vattanak, Ung Soviet, Florine van Driessen, Aaryan Dahal, Abhijit Mishra, Rupam Tripura, James J. Callery, Arjen M. Dondorp, Thomas J Peto, Phaik Yeong Cheah, Bipin Adhikari

**Affiliations:** a Mahidol-Oxford Tropical Medicine Research Unit, Faculty of Tropical Medicine, Mahidol University, Bangkok, Thailand; b National Centre for Parasitology, Entomology and Malaria Control, Phnom Penh, Cambodia; c School of Public Health, National Institute of Public Health, Phnom Penh, Cambodia; d Provincial Health Department, Stung Treng, Cambodia; e Centre for Tropical Medicine and Global Health, Nuffield Department of Medicine, University of Oxford, Oxford, UK

**Keywords:** Research, youth engagement, community engagement, Cambodia, capacity building

## Abstract

**Background:**

The Youth Advisory Group on Health and Research Engagement (YAGHRE) has been a collaborative initiative between school students and Mahidol Oxford Tropical Medicine Research Unit (MORU) staff in Siem Pang, Cambodia since 2021. Members provide input on health and research activities conducted by MORU.

**Objective:**

The main objective of this study was to explore key stakeholders’ perspectives on the YAGHRE.

**Methods:**

A total of 35 respondents participated in this study, including YAGHRE members (*n* = 14), teachers (*n* = 6), healthcare staff (*n* = 2), school students (*n* = 6), and family members (*n* = 7). Data were collected through focus group discussions and individual interviews. Thematic analysis was used to identify cross-cutting patterns in participants’ understandings, experiences, and recommendations related to YAGHRE.

**Results:**

YAGHRE members were viewed as a link between MORU and the community for health promotion and engagement with research. Participation in YAGHRE seemed to foster members’ confidence, communication, and digital literacy. Parents and teachers also reported improvements in members’ leadership and youth-led engagement activities. The knowledge and skills acquired were shared with families, peers, and community members, potentially enhancing awareness of health and hygiene. The participatory nature of YAGHRE, where members planned activities with MORU staff seemed to have cultivated a sense of ownership. YAGHRE was perceived as a transformative platform for youth skill-building and health education, strengthening community–research partnerships and local health and research capacity in rural Cambodia.

**Conclusions:**

Our engagement model suggests how community-based research and engagement can simultaneously advance scientific goals, build local capacity, and strengthen public trust through participatory approaches.

## Paper Context

- **Main findings:** Participation in YAGHRE strengthened youth confidence, skills, and leadership while enhancing community research relationships and local health awareness in rural Cambodia.
- **Added knowledge:** This study provides new evidence on how youth-led, participatory engagement can build health and research capacity in remote, resource-limited settings.
- **Global health impact on policy and action:** The YAGHRE model demonstrates how integrating youth advisory groups into research programs can promote equitable participation, strengthen trust, and guide community-engaged global health strategies.

## Background

Community engagement is described heterogeneously in the literature and is broadly defined as a collaborative process of working with groups of people connected by geographic location, shared interests, or common concerns to address issues that affect their well-being [1–3]. Community and public engagement are increasingly being promoted globally, more so recently because of the widening gaps and divisions between science and the public which ultimately can create two factions in society antagonizing each other [4,5]. If the growing gap between science and the public is not addressed through effective communication and engagement, public trust in scientific institutions may erode, leading to increased scepticism and resistance to science [6–8]. There are already emerging warning signs of how the public have become sceptical of science and its products and have been made much visible during the current COVID-19 pandemic [9,10]. Over the last few years (dominated by digital media) and more prominently during the pandemic, narratives against science, and its products are increasing, and are evidenced by the increase in alternative (conspiracy) theories about COVID-19, falsified information on cures for diseases – also referred to as the ‘infodemic’ [11,12]. The impacts are clear, for instance, low vaccine uptake in high-income countries together with demonstrations against the government on the imposition of public health measures and vaccination [13]. Literature around these concerns is also increasing, and echoes not just the widening gaps between science and the public, but the increasing antagonism against science [4,10,14,15].

While the COVID-19 pandemic has exposed the gaps and antagonism between science and the public, refusals and their aftereffects have long existed and have shown how precariously science and its uptake are progressing [5,11]. Undoubtedly, investing in promoting science translation through engagement with lay people (community or public or youth) is one important solution to mitigate the public-science gaps [16]. Efforts towards reducing the gaps are being implemented by major research institutions including translating research and science to the lay public in public venues [16]. Public events such as science café events and ‘pint of science’ events are increasingly being promoted in academic institutions where scientists are encouraged to engage with the public, share their knowledge, rectify misconceptions around science and ultimately increase trust and relationships with the public who are the end-users of science and its outcomes [17]. The idea of encouraging scientists to engage with the public carries nuanced and multifaceted benefits, underscoring their social responsibility as bearers of knowledge – not least the recognition that such engagement is inherently worthwhile, regardless of its measurable outcomes [18].

One important method to connect with the public is through the youth in society, who can be the bridge between scientists and the adult public and are potential agents of social change [19]. Apart from youth being end-users of science, they are also an excellent conduit between scientists and wider community members because of their enthusiasm to learn and share new knowledge [19]. The United Nations Educational, Scientific and Cultural Organization (UNESCO) has been engaging youth groups since 1999 to discuss the development of their communities, foster peace, alleviate poverty, and inequity [20]. This also aligns with the recent WHO – UNICEF – Lancet Commission on ‘a future for the world’s children? which has launched a call for the involvement of young people in all decision-making policies and coalitions [21,22]. Literature around public engagement has shown how engaging school students who have a basic orientation to science in school are an important part of the society whose diligent engagement can have manifold positive consequences [23,24].

In June 2021, the Mahidol-Oxford Tropical Medicine Research Unit (MORU) in partnership with the Cambodian National Malaria (CNM) program established the Youth Advisory Group on Health and Research Engagement (YAGHRE). YAGHRE members aged between 16 and 22 were selected from a local school in Siem Pang based on their interest and recommendations from their schoolteachers. The objectives of YAGHRE are (1) to create a sustainable network of young students; (2) to build students’ capacity in presentation, communication, and leadership skills; (3) to build knowledge of common diseases and public health problems; (4) to build knowledge of the basics of health research and ethics; (5) to improve community health through health education; and (6) to identify and better understand community health issues and concerns related to research participation. For instance, in a recent engagement activity on antimicrobial resistance (AMR), YAGHRE members provided input into research protocols and engagement materials, particularly on comprehensibility and practical aspects, co-creating the health messages [25,26].

Our 2024 reflective analysis [27] identified five interrelated outcomes that illustrate the broader impact of YAGHRE activities: (1) increased respect, where co-developed activities appeared to foster more reciprocal and context-sensitive interactions between stakeholders; (2) built trust and relationships, with repeated engagement and sustained interactions contributing to the development of trust and social connectedness; (3) improved health and research literacy, as participatory learning approaches supported a deeper understanding of health and research processes; (4) improved uptake of health and research interventions, suggesting that improved communication and engagement may have facilitated participants’ willingness to engage with interventions; and (5) improved community health, indicating that these combined processes may have contributed to broader, incremental health benefits at the community level.

The primary objective of this study was to explore the perspectives on YAGHRE activities and their implications among YAGHRE members, and stakeholder associated with it including teachers, healthcare staff, school students and their family members.

## Methods

### Study design

This was a descriptive qualitative study. The study followed the Consolidated Criteria for Reporting Qualitative Research (COREQ) guidelines (Supplementary file 1) [28].

### Study setting

The study was conducted in Siem Pang, one of the remotest districts of Cambodia where MORU’s research clinic was stationed to conduct malaria research [27]. The Youth Advisory Group on Health and Research Engagement program was deliberately selected for Siem Pang district (in contrast to prior research sites within Cambodia) because of its remote location, high malaria incidence, and the overall deprivation experienced by its population, to ensure that these remote communities are represented in MORU’s research and engagement portfolio. Based on a 2019 census, the population of Siem Pang is a little over 25,000 people, spread across an area of 4,561 km^2^ in 28 villages [29].

With the majority of the population comprising ethnic minorities, forest goers, and agricultural or farm workers, average daily wages and living conditions are extremely difficult in the district [30]. Forty-one primary schools (grades 1–6), five secondary schools (grades 7–9), and one high school (grades 10–12) provide formal education in Siem Pang, although limited resources and logistical challenges remain major barriers to quality, access, and attendance. Based on our own interaction among those who have participated in MORU’s study, the majority of the adult population is illiterate and has never attended school. In addition, social, cultural, and linguistic diversity affects community practices (e.g. festivals, interactions), including the uptake of health services.

### Study participants

**Figure 1.**
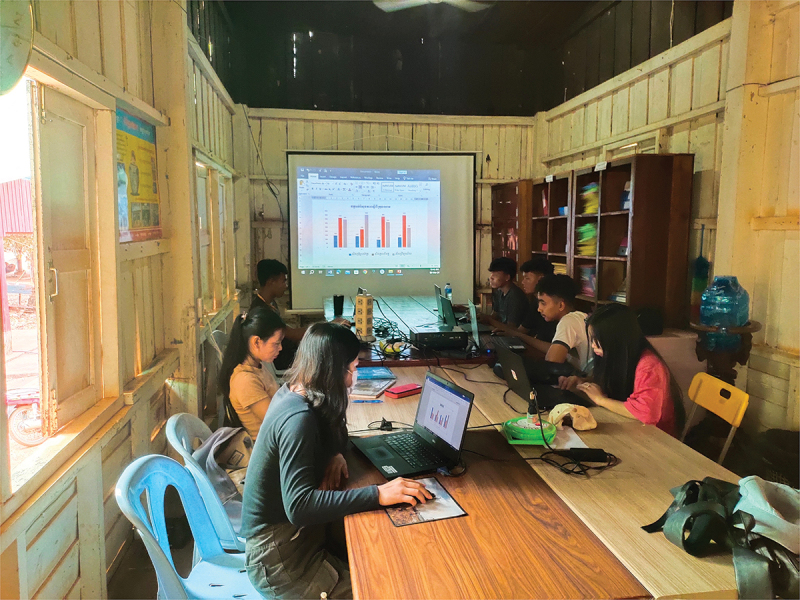
YAGHRE members participating in engagement activities at MORU’s office.

Our sampling approach was based on the basic tenet of qualitative research, where respondents were selected based on their role, contribution, and lived experience related to YAGHRE activities (Figure 1) [31]. A total of 35 respondents participated in this study between June and August 2025 including 14 YAGHRE members (current and former), six school students who were not members of YAGHRE, six teachers, two health care staff, and seven family members of the YAGHRE members (Table 1). The number of respondents for this study was based on the principles of ‘data saturation’, that is data were collected until no new data/themes emerged from further interviews [32]. None of the approached persons who were asked to participate in the interview refused. The inclusion criteria included any relevant individual aged 16 years or older; respondents from any of the categories of stakeholders listed above who were associated with/had knowledge of YAGHRE; and participants who were willing and able to provide informed consent for an interview and could communicate without difficulties. ME, a female health worker, and HV, a male health worker, both local staff who founded and supervised YAGHRE, conducted the interviews and FGDs jointly with all respondents in this study. Both ME and HV were trained and supported by BA, a male Nepalese MD specializing in social science and qualitative research, with more than a decade of experience.

**Table 1.** Socio-demographics of the respondents of the study.

Study Participants	Interview/discussion ID	Age	Sex	Education level	Occupation
YAGHRE members	YAGHRE-01	23	M	Bachelor degree	Primary school teacher
	YAGHRE-02	22	F	Grade 12	Primary school teacher
	YAGHRE-03	25	F	Bachelor degree	Employee (NGO)
	YAGHRE-04	22	F	Bachelor degree	Second year student
	One FGD (YAGHRE-05)	23	F	Bachelor degree	Second year student and employee (NGO)
		21	M	Grade 12	Primary school teacher
		20	M	Grade 12	Primary school teacher
		21	M	Grade 12	Credit officer of Microfinance
		18	F	Grade 12	YAGHE member
	One FGD (YAGHRE-06)	21	F	Grade 12	Construction material Seller
		18	F	Grade 12	YAGHE member
		20	F	Grade 12	YAGHE member
		22	F	Bachelor degree	Second year student
		19	M	Grade 12	YAGHE member
Community members	PE-CM-01	28	M	Bachelor degree	Secondary school teacher
	PE-CM-02	27	M	Bachelor degree	Secondary school teacher
	PE-CM-03	29	M	Bachelor degree	High school teacher
	PE-CM-04	31	F	Bachelor degree	Secondary school teacher
	PE-CM-05	37	M	Nurse’s degree	Nurse of Santepheap health center
	PE-CM-06	40	M	Grade 2	Farmer & Seller
	PE-CM-07	30	M	Bachelor degree	Secondary school teacher
	PE-CM-08	34	M	Bachelor degree	High school teacher
	PE-CM-09	54	M	Grade 4	Commune council officer & Farmer
	PE-CM-10	40	M	Grade 12	District council officer & Farmer
	PE-CM-11	84	M	Grade 12	Farmer
	PE-CM-12	35	F	Grade 12	District officer
	PE-CM-13	54	M	Grade 6	Employee (NGO) & Farmer
	PE-CM-14	45	M	Grade 4	Famer
		41	F	Grade 1	Farmer
	PE-CM-15	52	M	Nurse’s degree	Chief of Siem Pang health center
	One FGD (PE-CM-16)	18	M	Grade 10	High school student
		18	M	Grade 11	High school student
		20	M	Grade 11	High school student
		18	F	Grade 10	High school student
		19	F	Grade 11	High school student
		18	F	Grade 11	High school student

**Figure 2.**
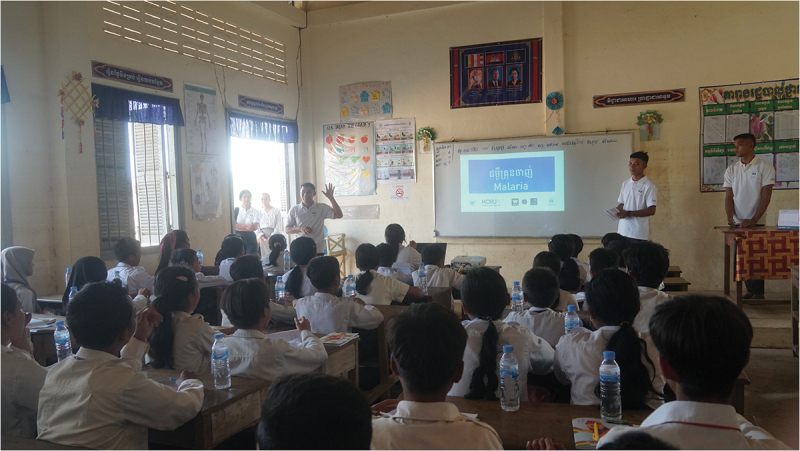
YAGHRE members engaging with school students in Siem Pang.

**Figure 3.**
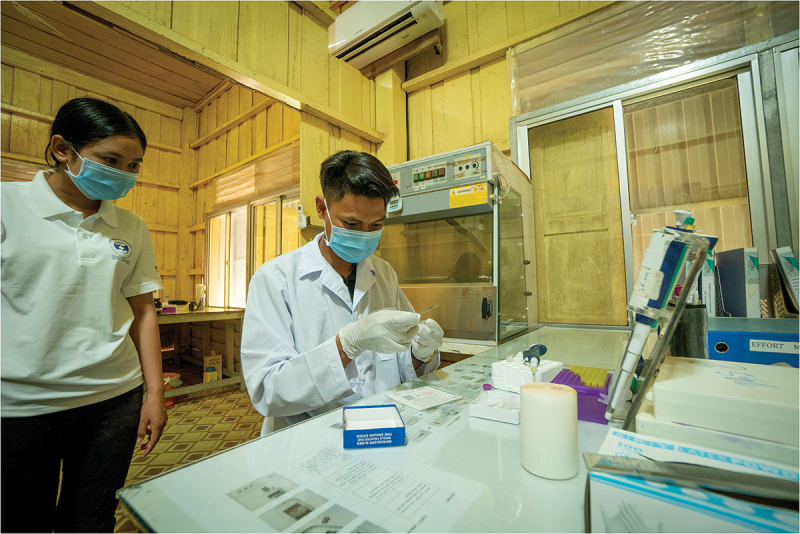
YAGHRE members’ involvement in MORU’s laboratory.

**Figure 4.**
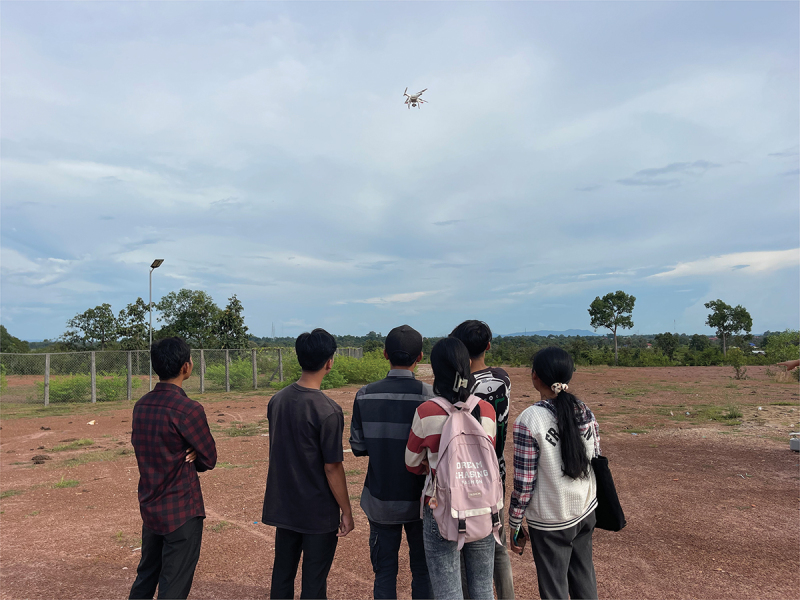
YAGHRE members learning how to fly a drone.

ME and HV knew the YAGHRE members, and some of the health workers, but not the students, and family members. Aware of the relationship between the researcher and some of the respondents, interviewers encouraged them to reflect on non-personal implications of YAGHRE. Soon after approaching a potential respondent, a written informed consent was collected before the interviews. A separate written media informed consent form was obtained from all individuals featured in the engagement activities captured in photographs (Figures 1–4) for publication of their images. The study was carried out in accordance with the principles of the Declaration of Helsinki. All approached potential respondents agreed to participate after the appointment was made at a time and venue convenient for the respondents.

### Data collection and interview guides

A semi-structured interview (SSI) and focus group discussion (FGD) guides were constructed to explore the perspectives around engagement activities from YAGHRE members (Supplementary file 2) and the stakeholders (Supplementary file 3). SSI/FGD guides were prepared and adapted to interview each of the types of respondents. SSI/FGD guides were constructed based on the overarching themes related to the scholarship of community engagement and these themes were further refined based on the discussion among the team and YAGHRE members, and during the data collection. The themes included (1) knowledge on YAGHRE, (2) benefits and drawbacks of YAGHRE, (3) participatory aspects of YAGHRE and finally (4) recommendations for YAGHRE. SSI/FGD guide were first pre-tested by interviewing researchers and few relevant stakeholders to ensure the questions and themes were pertinent to the research objective.

The interviews were conducted face to face with all potential respondents based on the SSI/FGD guide and each interview lasted around 45–60 minutes and were recorded on an audio-recorder. All interviews were conducted in Khmer with a few, real-time translation in English and Khmer, specifically when conducting FGDs (*n* = 2) in the presence of BA. Interviews/discussions were conducted at MORU’s clinic and respondents were reimbursed and compensated for their time. All interviews were transcribed verbatim into English. Post-interview discussions among the team were held to document the interpretations related to the interviews.

## Data analysis

All the transcripts and the interviewer’s notes were first cross-checked with the audio-recordings. Transcripts were analyzed using NVivo by BA in consultation with ME and HV, a qualitative software package used to generate a codebook and later synthesize themes. Six phases of thematic analysis outlined by Braun and Clarke [33] were followed that included: (1) Familiarizing with the data (transcribing data, reading and rereading the data, noting down initial ideas); (2) Generating initial codes (coding interesting features of the data systematically across the entire data set, collating data relevant to each code); (3) Searching for themes (collating codes into potential themes, gathering all data relevant to each potential theme); (4) Reviewing themes (checking if the themes work in relation to the coded extracts and the entire data set, generating a thematic map); (5) Defining and naming themes (ongoing analysis to refine the specifics of each theme, and the overall process of the analysis); and (6) Producing the report (selection of vivid, compelling extract examples, final analysis of selected extracts, relating back to the research question and literature, producing a scholarly report of the analysis) [33,34].

To address the research questions, themes and the supporting quotes were presented as the findings of this study.

## Results

YAGHRE was perceived to serve as a bridge between MORU and the local community, engaging young people in health promotion and learning. Insights from those who were YAGHRE members and those who were not, offered a range of perspectives on how YAGHRE activities were perceived in the school, community and at health centres. Together with their reflections and experiences of YAGHRE over the last four years, the major themes from this study are presented below.

### Understanding of YAGHRE’s purpose and formation

Our interviews and FGDs revealed that YAGHRE members demonstrated a clear understanding of the group’s objectives and the collaboration underpinning its creation. For most members, YAGHRE was seen as a bridge between MORU and the local community, designed to engage youth in health promotion and health information dissemination, including engaging with the fellow students and community members.

For me, the Youth Advisory Group on Health and Research is a collaboration between the MORU organization and the staff at the Siem Pang Health Center and the MORU staff in studying and researching diseases and spreading the information to students in schools or to the communities. (YAGHRE-05, FGD among five members)

In contrast, parents, teachers, fellow students and health authority members had more varied interpretations of YAGHRE’s purpose. While parents often understood it through their children’s participation, their perceptions emphasized the group’s educational and preventive health roles rather than its broader functions, such as supporting research and offering feedback in engagement initiatives of MORU.

Yes! I heard it from my daughter who came to study with MORU, so when she got home, she told us. It means that she understands about any diseases and how to prevent them clearly. (PE-CM-10, 40 years old, male)

This distinction reveals the multiple meanings YAGHRE holds within the community. While youth viewed it as a learning platform, parents perceived it as an opportunity for their children to acquire valuable life and health knowledge.

### Perceived benefits and capacity development

Across the respondent groups, there was consistent recognition of the personal and social benefits emerging from YAGHRE’s activities. Parents often highlighted the tangible improvements they observed in their children’s confidence, public speaking, and technological literacy, noting that the program provided opportunities otherwise unavailable in rural Siem Pang.

What interests me most about MORU is about the activities, as MORU provides opportunities for young people to develop their skills … For some people, they may not be very good at learning, but they are capable of doing other things. Obviously, like he works with MORU, I see that he is capable of making presentations, is capable of teaching students, is capable of using various technologies, he knows how to use Microsoft word, excel, and PowerPoint. (PE-CM-01, 28 years old, male)

Parents also identified how YAGHRE’s influence extended beyond individual development and employability, particularly through learning and engaging with community members on health issues that most affected the community.

I believe that they will have a better chance of finding jobs than those who have not been with MORU. First, they developed their own abilities. Second, they shared good knowledge with young people as well as with other villagers to know about health issues, health care, so that their lives are healthy. (PE-CM-01, 28 years old, male)

These perspectives highlight how YAGHRE operated simultaneously as a platform for youth skill-building and as a mechanism for community health promotion.

Teachers further reinforced this view, observing marked improvements in students’ confidence, leadership, and presentation skills (Figure 1) following their participation.

Some of them were not very good at speaking when they were in school. When they joined the MORU organization, they were able to express themselves in educational activities, speak clearly, and explain the meaning of the content correctly. Some youths were shy before they joined the MORU organization, but now they are brave and capable of administrative work. (PE-CM-08, 34 years old, male)

Several YAGHRE members themselves described the practical competencies they had acquired through participation, especially in computing, presentation, and communication – skills that translated directly to their education and employment contexts.

The activity I like the most is related to presentations … when I joined the MORU organization, MORU taught me how to communicate, make presentations, and how to teach. They also encourage me to be brave and also taught me to use computer … When I started working, like I’m a teacher now, I went to school and would use those skills regularly. (YAGHR member-01, 23 years old, male)

It really helped me a lot because studying with MORU … I did not even know how to use a keyboard before. However, when I joined and learned with MORU, I gained a lot of knowledge … I already know how to make reports, posters, and PowerPoint slides. (YAGHRE member, 25 years old, female)

These activities reveal how YAGHRE fostered multidimensional learning, that is, technical, communicative, and interpersonal, creating a generation of young people with enhanced confidence and employable skills.

### Community knowledge transfer and activities

A strong pattern across interviews was the intergenerational flow of health information from youth members to their families and wider communities. Parents consistently recounted how their children shared key public health messages, illustrating the sharing of knowledge beyond the immediate training environment.

He [my son] told me that I should sleep inside the mosquito nets at night to prevent mosquitoes from biting us. We might get sick from the mosquitoes and this disease will be spread from one person to another … . My children have told me about how to wash our hands and feet before eating, and how to prevent the diseases from affecting our bodies … They say that diseases will spread to our bodies, and we should wash our hands with soap and water before eating. (PE-CM-06, 40 years old, male)

Parents often described these exchanges with pride, interpreting them as signs of both moral and intellectual growth in their children. Across all parents, gratitude towards MORU and the staff offering such a platform was common.

Teachers and community members likewise valued YAGHRE’s outreach, describing how youth-led presentations enhanced school-based health education (Figure 2) and stimulated greater community participation. Some teachers regarded YAGHRE’s engagement on health topics with their students to be superior (better), partly because the presentations were often prepared for weeks and months for the content and the entertainment values they brought together.

Sometimes during class, I told them that I will invite the MORU [YAGHRE members] to come and teach them, because when I teach them, they don’t pay attention and listen to me like when you [YAGHRE members] come. (PE-CM-04, 31 years old, male)

They go to spread the word in schools, secondary schools, and high schools about ways to prevent diseases and other illnesses to students, and they distribute leaflets for them to read and share with their parents. (PE-CM-10, 40 years old, male)

These findings highlight YAGHRE’s role as an interface between formal health education and community practice, facilitating participatory learning and expanding the reach of public health messaging.

### Participatory engagement and co-decision making

The participatory nature of YAGHRE was evident in how youth members described their involvement in planning and decision-making. Members’ initial visits to the laboratory (Figure 3), and bedside clinics of MORU gradually seemed to have increased their familiarity with the research areas, mostly focused on malaria and infectious diseases. Apart from inherent values related to the sharing of time and space at MORU and its staff, members gradually learnt how this contradicted with the typical didactic methods of learning at their school. Rather than being passive recipients of instruction, members reported active collaboration with MORU staff in selecting topics and organizing outreach.

Some topics are decided by the MORU, some are selected by the youth group, based on the requests of the school students and on actual problems that occur in the community. (YAGHRE member-03, 25 years old, female)

For me, the decisions were made jointly, 50% by MORU staffs and 50% by the youth volunteer group … we coordinate with each other and choose a day when everyone is available. (YAGHRE-05, FGD with five members)

Such participatory processes may have contributed to members’ sense of ownership over health topics including planning for wider engagement. This process apparently also reinforced their confidence and civic responsibility in the community. The inclusion of locally relevant topics such as hygiene, malaria prevention, and in some cases, reproductive health demonstrated both YAGHRE and MORU’s responsiveness to community needs.

Those who decided to add the activities … are the youth group and the staff of MORU. Because the youth group has rights and can make decisions, such as some lessons that MORU does not have in study or research, such as diabetes, reproductive health etc. (YAGHR-06, FGD with five members)

This co-production approach positioned YAGHRE as a participatory learning space, bridging institutional structures with youth-led initiatives and enhancing contextual relevance.

### Recommendations for future engagement

Participants across groups including teachers, parents, health authorities, and youth members expressed strong support for the continuation and expansion of MORU’s activities in the Siem Pang district. Their recommendations centered on sustaining YAGHRE’s impact, broadening youth participation, enhancing local health education, and extending MORU’s research presence to address emerging health priorities.

Teachers and parents consistently recommended that MORU continue and expand the YAGHRE initiative, emphasizing its educational, social, and developmental value for local youth. They viewed YAGHRE as a platform for building confidence, digital literacy (Figure 1 and 4), and leadership among students, while strengthening community health knowledge (Figure 2). Aware of the recent decline in malaria, as MORU was ending some of the malaria clinical trials, YAGHRE members expressed their desire for the continuation of YAGHRE activities. Some even recommended expanding the number of youth members recruited in each batch, with some offering ideas to expand the inclusion of younger students (other than the current inclusion criteria of grades 11 and 12).

I have a suggestion if the project still continues. I suggest that if it is possible to take grade 9 students to be a youth group [member]. I think it would be great because I also want my students to participate. If they could participate in this training, they will be more courageous. (PE-CM-07, 30 years old, male)

Just a request is that we want the MORU organization stay in Siem Pang for long time to continue recruiting young students to volunteer as health and research consultants and continue to spread the word to other schools and communities in Siem Pang district. (PE-CM-08, 34 years old, male)

Participants also highlighted the importance of maintaining YAGHRE’s focus on health education and practical skills. Teachers recommended expanding the number of trainees each year and including new topics, such as non-communicable diseases and digital health. These recommendations strongly reflect their desire to institutionalize YAGHRE as a long-term community asset, one capable of nurturing youth leadership and sustaining grassroots health literacy.

## Discussion

This study shows that YAGHRE operates as a bridge between research institutions and rural communities by positioning youth as active agents in health promotion and engagement. Participation enhanced members’ confidence, communication, and leadership, while also facilitating the spread of health knowledge to families and community networks. The participatory design enhanced ownership and sustained engagement. These findings highlight the potential of youth-led platforms to strengthen local capacity, community–research relationships, and trust in research in resource-limited settings.

The Youth Advisory Group on Health and Research Engagement combines both intrinsic and extrinsic properties of community engagement [27]. Intrinsic properties of CE are often inductive serving those who are engaged to enhance their agenda, for example, skill sets and capacity of the youth members and health and hygiene of the community [2,27]. Extrinsic properties of CE often support deductively to ensure that research conducted is ethical, participants feel respected, and their (unobvious) vulnerabilities are safeguarded [2]. Both of these properties, without one being superior to the other are critical features of CE and are addressed by the YAGHRE activities in Siem Pang, Cambodia.

The findings demonstrate that YAGHRE functioned as a transformative participatory model that values youth as important agents of the community [35], fostering community health literacy, and local collaboration [36,37]. Youth participants shared gaining practical skills and self-confidence, while parents and teachers perceived tangible benefits at both personal and community levels. The multi-directional flow of knowledge, from MORU to youth, and from youth to the community and vice versa illustrates how such an engagement model can catalyze health messaging and health promotion in resource-limited settings [38]. Indeed, these activities have multiplicative implications in strengthening the institutional trust of a research organization while also serving as a learning platform for MORU’s researchers [39,40]. Trust and relationship are often intangible features emanating from engagement, including maintaining ‘presence’ over a period of time, and thus YAGHRE and similar activities have a critical role in promoting research and institutional credibility [41,42]. YAGHRE is continuing in Siem Pang despite the completion of malaria clinical trials, reinforcing the intrinsic purpose of engagement.

Respondents shared several benefits of YAGHRE, that ranged from individual benefits to the members and subsequent benefits to the community [43]. Some of the skill sets these youth members developed were often found useful in their higher education. Members were able to easily differentiate their own standing at higher education institutes and could not resist seeing the difference between their fellow colleagues. Career-related benefits have been shown to be one of the most valued attributes in Kenya where Davies’ longitudinal follow-up on students trained at KEMRI later adopted careers in science [44,45]. Some of these gains had other intangible benefits such as leadership skills, confidence, and in general their ability to explore and synthesize knowledge on the topics they were interested in [46,47]. Building the skills and capacities of youth can serve as a sustainable contribution to society – one that extends beyond the immediate, cross-sectional impacts of engagement. This has clear pedagogical implications for educational institutions, particularly schools and campuses, in integrating and adapting YAGHRE training and engagement materials into curricula and activities for younger populations.

The research and health-related knowledge gained by YAGHRE members were often shared among their parents and community members thus multiplying health-related knowledge in reaching to wider circles and imparting benefits beyond the trainees [38]. Several scholars associate this characteristic of engagement with the diffusion of knowledge, whereby dissemination processes help promote understanding among the public [48,49]. However, the translation of knowledge into practice is often non-linear and shaped by interests, agencies, and multiple social, contextual, and cultural factors [50,51]. Engagement itself could be viewed as a two-way learning process, where all participants benefit from sharing their knowledge.

Engagement initiatives with YAGHRE were deemed highly participatory, as demonstrated by how the agenda for engagement was shaped by suggestions from the members, including their recent co-creation of AMR-related engagement activities in districts across Cambodia [25,26]. Such a participatory approach has been highlighted to address power sharing with communities, echoing equity focused studies across borders [39,52]. While their contributions to research and public engagement were valuable, most YAGHRE activities involved joint decisions on health topics, with youth members proposing issues identified through their school and community health priority exercises [53]. Such a method of embracing health topics suggested, demonstrates inductive or bottom-up methods of engagement which address the epistemic balance between ‘etic’ and ‘emic’ perspectives while also tailoring and serving the concerns of the local communities directly [52,54–56]. In recent years, priority setting exercises based on community feedback have gained traction and respect and address community’s concerns rather than a conventional vertical approach [53,57–59].

Across groups, participants emphasized continuity, expansion, and integration of the YAGHRE program, including younger cohorts and expanding the disease scope. Embedding engagement across the most remote and deprived communities in Cambodia also aligns with global health discourse by proactively ensuring equity in research, health, and capacity benefits through engagement [3,52]. Participants’ convergent suggestions to ensure collaboration with communities and the extension of MORU’s projects to address wider health challenges also highlight the need to be responsive to community suggestions, for global health equity [54]. Advancing equity in global health research requires power-conscious, socially and culturally aware approaches that enable informed agents to mobilize meaningful discourses and actions [52,54,56,60]. These recommendations underscore the strong local endorsement of MORU’s participatory model and its perceived value as both an educational and public health catalyst in remote Cambodian settings.

## Strengths and limitations

The study interviewed YAGHRE members (both current and former) and their parents, in addition to teachers and health authorities who may have been appreciative of the general engagement activities, incurring desirability bias in their responses. Authors also reflected on why respondents may have been less likely to critique the engagement activities, given cultural norms of modesty and conformity in Cambodia. Nonetheless, this study included respondents who were not YAGHRE members such as school students, teachers, health workers, and parents who did not have direct affiliation. Respondents were also asked to reflect on limitations of the activities, and thus also yielded some recommendations for the future of YAGHRE activities. First, all YAGHRE activities occurred within the environment of MORU, where clinical trials were often the main studies, their exposure and contribution to overall studies may have promoted their understanding of the research related to malaria more than other diseases. Second, YAGHRE members were engaged in batches (*n* = 4), some of the older batches who were already employed or were attending university may also have influenced the younger batches. Lastly, although Siem Pang’s population is ethnically and linguistically diverse, YAGHRE members were fluent in Khmer, the official language used in educational institutions. Nonetheless, complex topics were often discussed and rehearsed over multiple sessions before members felt confident explaining them in local ethnic languages, particularly in remote villages with low literacy levels.

## Conclusions

YAGHRE in Siem Pang exemplifies how youth-led initiatives can integrate intrinsic and extrinsic dimensions of community engagement. Aware of the background remoteness and resource limitations of the site, YAGHRE may also have enhanced equity-focused approaches within engagement. By fostering skills, confidence, and local agency, it upskilled participants while reinforcing ethical, trust-based relationships between researchers and communities. Through participatory planning and knowledge sharing, YAGHRE bridged youth’s capacity development, community health education, and ultimately research credibility, illustrating how sustained, locally grounded engagement can advance both community well-being and institutional trust.

## Supplementary Material

Supporting Information File 2.docx

Supporting Information File 3.docx

Supporting_Information_File_1_clean.docx

## Data Availability

Because of the nature of the qualitative data in this study, even if the data are anonymized, potential respondents are identifiable. Both MORU and the local ethics committee restrict the sharing of data that can potentially identify the respondents. The data are available upon request to the Mahidol Oxford Tropical Medicine Research Unit Data Access Committee (datasharing@tropmedres.ac) complying with the data access policy on a case-by-case basis (https://www.tropmedres.ac/units/moru-bangkok/bioethics-engagement/data-sharing/moru-tropical-network-policy-on-sharing-data-and-other-outputs).

## Abbreviation

AMR: Antimicrobial Resistance
CE: Community engagement
COREQ: Consolidated Criteria for reporting qualitative research
COVID-19: Coronavirus Disease-2019
FGD: Focus Group Discussion
MORU: Mahidol-Oxford Tropical Medicine Research Unit
OxTREC: Oxford Tropical Medicine Research Ethics Committee
SSI: Semi-structured interview
UNESCO: United Nations Educational, Scientific and Cultural Organization
YAGHRE: Youth Advisory Group on Health and Research Engagement.
