# Supplementary material for: Engaging youth in health and research in rural Cambodia: a qualitative study
Source: Glob Health Action. 2026 Jun 15;19(1):2684845. doi: 10.1080/16549716.2026.2684845 (PMC13270867; doi:10.1080/16549716.2026.2684845)
Supplement: Supporting Information File 3.docx [file ZGHA_A_2684845_SM2972.docx]

# Supplementary File 3: SSI/FGD guide for community members (school students, teachers and healthcare workers)

# Engaging youth in health and research in rural Cambodia: a qualitative study

Mom Ean^1^**^†^**, Lek Dysoley^2,3^**^†^**, Hem Vattanak^1^, Ung Soviet^4^, Florine van Driessen^1^**,** Aaryan Dahal^1^, Abhijit Mishra^1^, Rupam Tripura^1,5^, James Callery^1,5^, Arjen Dondorp^1,5^, Tom Peto^1,5^, Phaik Yeong Cheah^1,5^, Bipin Adhikari^1,5*^

^1^Mahidol-Oxford Tropical Medicine Research Unit, Faculty of Tropical Medicine, Mahidol University, Bangkok, Thailand

^2^CNM National Centre for Parasitology, Entomology and Malaria Control, Phnom Penh, Cambodia

^3^School of Public Health, National Institute of Public Health, Phnom Penh, Cambodia.

^4^Provincial Health Department, Stung Treng, Cambodia

^5^Centre for Tropical Medicine and Global Health, Nuffield Department of Medicine, University of Oxford, Oxford, UK

**^†^Equal** contributors

[*Bipin@tropmedres.ac](mailto:*Bipin@tropmedres.ac)

**Journal**: Global Health Action

- Use this interview guide for interviews with health workers (

| **SSI/FGD number:**  **Location:** | | | | | |
| --- | --- | --- | --- | --- | --- |
| **SSI/FGD Note Takers Sheet** | | | | | |
| **Note Takers initials:** | | | | | |
| **Date: DD-MM-YYY** | | | | | |
| **Dispensary location (town/settlement/neighbourhood):** | | | | | |
| **Socio-demographics** | | | | | |
| **Interviewer ID** | **Age** | **Sex** | **Education (years)** | **Remarks** | **Respondent ID** |
|  |  |  |  |  |  |
|  |  |  |  |  |  |
|  |  |  |  |  |  |
|  |  |  |  |  |  |
|  |  |  |  |  |  |
|  |  |  |  |  |  |
|  |  |  |  |  |  |
|  |  |  |  |  |  |

- Avoid using closed questions and probe frequently
- **Obtain demographic data in the sheet below.**

1) Explain the study and ask for if the respondent(s) is/are ready

*e.g “Thank you for seeing me today…are you happy to take part in this study?*

**Turn the recorder on**

*Ok, so I have turned the microphone on. I just wanted to ask again, are you happy to take part in this study by speaking with me today?*

2) Explore the themes below:

| **Topic** | **Suggested questions/probes** |
| --- | --- |
| **Tell me about your background** | - What do you do? - Where do you study? - What is your school routine like? - How is your routine at the home? |
| **About YAGHRE** | - Have you heard of YAGHR? - Do you have friends as members of YAGHR? - What do you think of YAGHRE activities? - Are there specific activities that you like and why? |
| **Benefits of YAGHR activities** | - Do you see any benefits of YAGHR activities and if so why? - Do you think these activities have any impacts on you? if so what are they? (e.g. any new knowledge) - Do you think YAGHRE activities have impacts on community? If so what are they? - Would you have joined as a member of YAGHRE? If so why? |
| **Disadvantages of YAGHR activities** | - Can you think of any inconveniences due to YAGHRE? And why? |
| **Recommendations for YAGHR** | - Would you like to recommend any new activities current YAGHRE? - What can be improved and how? |
| **CLOSING**  Summarise the key points from the interview | Do you have any questions or concerns you’d like to raise? Thank you. |
